# Transcriptomic Analysis of Leaf Sheath Maturation in Maize

**DOI:** 10.3390/ijms20102472

**Published:** 2019-05-19

**Authors:** Lei Dong, Lei Qin, Xiuru Dai, Zehong Ding, Ran Bi, Peng Liu, Yanhui Chen, Thomas P. Brutnell, Xianglan Wang, Pinghua Li

**Affiliations:** 1 College of Agronomy, Synergetic Innovation Centre of Henan Grain Crops and National Key Laboratory of Wheat and Maize Crop Science, Henan Agricultural University, Zhengzhou 450002, China; dongleixyz@126.com (L.D.); chenyanhui@henau.edu.cn (Y.C.); tom@viridisgenomics.com (T.P.B.); 2 State Key Laboratory of Crop Biology, College of Agronomic Sciences, Shandong Agricultural University, Tai’an 271018, China; qinlei2012shandong@163.com (L.Q.); daixiuru1993@163.com (X.D.); 3 The Institute of Tropical Bioscience and Biotechnology (ITBB), Chinese Academy of Tropical Agricultural Sciences (CATAS), Haikou 571101, China; dingzehong@itbb.org.cn; 4 Department of Statistics, Iowa State University, Ames, IA 50011, USA; biran@iastate.edu (R.B.); pliu@iastate.edu (P.L.)

**Keywords:** leaf sheath, maturation, transcriptional dynamics, transcriptome

## Abstract

The morphological development of the leaf greatly influences plant architecture and crop yields. The maize leaf is composed of a leaf blade, ligule and sheath. Although extensive transcriptional profiling of the tissues along the longitudinal axis of the developing maize leaf blade has been conducted, little is known about the transcriptional dynamics in sheath tissues, which play important roles in supporting the leaf blade. Using a comprehensive transcriptome dataset, we demonstrated that the leaf sheath transcriptome dynamically changes during maturation, with the construction of basic cellular structures at the earliest stages of sheath maturation with a transition to cell wall biosynthesis and modifications. The transcriptome again changes with photosynthesis and lignin biosynthesis at the last stage of sheath tissue maturation. The different tissues of the maize leaf are highly specialized in their biological functions and we identified 15 genes expressed at significantly higher levels in the leaf sheath compared with their expression in the leaf blade, including the *BOP2* homologs *GRMZM2G026556* and *GRMZM2G022606*, *DOGT1* (*GRMZM2G403740*) and transcription factors from the B3 domain, C2H2 zinc finger and homeobox gene families, implicating these genes in sheath maturation and organ specialization.

## 1. Introduction

Maize (*Zea mays* L.) is one of the most important grain crops globally. It is not only used as a grain food and feed but is also preserved as corn silage and as a bioenergy source. Regardless of its final use, the accumulation of maize biomass is important. The leaf is the primary source of photosynthate; thus, its morphological development greatly influences plant biomass and yield. A typical mature maize leaf is made up of three parts: The leaf blade, the ligular region and the leaf sheath. The blade is the distal part of the maize leaf and the major organ for photosynthesis, the ligular region is a wedge-shaped structure connecting the leaf blade and leaf sheath that acts as a hinge to project the leaf blade away from the stem, and the sheath wraps around the stem and provides strength for the growth and development of the leaf blade. The shape and development of these three regions control the architecture of the maize leaf, which is important for photosynthesis and ultimately maize yields [1,2,3,4,5].

The development of grass leaves proceeds basipetally, with the distal cells differentiating and maturing first while the basal cells divide and expand. Several recent studies have portrayed a very dynamic biochemical differentiation process along the maize leaf blade. Here, basic cellular functions—such as DNA synthesis and cell wall synthesis—become enriched in the basal region of the leaf sink, which transitions to secondary cell wall biosynthesis and the establishment of the photosynthetic machinery in the source-sink transition zone and the dominant photosynthetic reactions in the distal part of the leaf [6,7]. Also, several mutants have been identified that influence the morphogenesis of the leaf blade in maize, e.g., *ns* (narrow sheath) and *rld1* (rolled leaf 1) [8,9], and produce abnormal leaf blade development in maize. Several genes that control ligule development in maize have been identified. These include *LIGULELESS1* (*LG1*), which encodes a Squamosa binding protein (SBP) family transcription factor; *LIGULELESS2* (*LG2*), encoding a basic leucine zipper (bZIP) family transcription factor; and *LIGULELESS NARROW* (*LGN*), a putative serine-threonine kinase that controls early ligule formation in maize leaves [10,11,12,13,14]. Compared to studies on the leaf blade and ligule tissues, very few studies have focused on the leaf sheath, and our understanding of sheath development is limited.

The leaf sheath wraps around the stem in maize and is believed to provide strength for the growth and development of the leaf blade. Hatfield and colleagues compared changes in the cell wall component in maize leaf blades [15], midribs and sheaths from nodes 9 to 14 and concluded that sheath and midrib tissues always accumulate more neutral sugars, lignin and total phenolics than blade tissues. These metabolite measurements may explain the higher mechanical strength of leaf sheaths; however, information at the molecular level is still limited.

Most mutations that affect the blade-sheath boundary appear to extend the sheath tissue into the blade, but the functions of genes that regulate the development of the sheath tissues are poorly understood, and almost no genes have been identified that distinguish the leaf sheath from the blade and ligule tissues. To investigate the transcriptional dynamics associated with the maturation of maize sheath tissues, we conducted RNA sequencing (RNA-seq) profiling experiments to capture the progressive stages of sheath maturation. These data reveal a dynamic transcriptional process during leaf sheath maturation. In particular, we have defined transcriptional regulators that may specify unique activities in sheath tissue relative to those in the leaf blade. This dataset serves as a foundation for future studies of maize sheath development and maturation.

## 2. Results

### 2.1. Defining the Leaf Sheath Transcriptome

**Figure 1:**
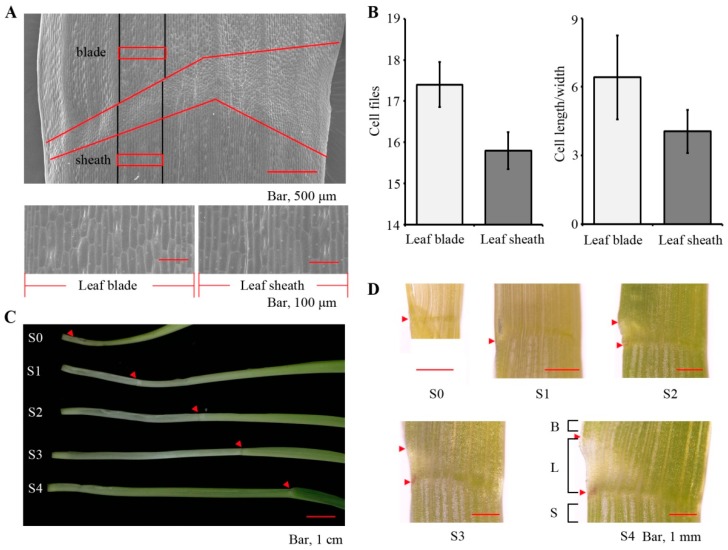
Observation of the leaf sheath and blade tissues. (**A**) Scanning electron micrograph of a mature maize leaf. B73 seedlings were grown for 13 days (stage 4). The red line indicates the ligular region, the tissues above the line are the blade and the tissues below the line are sheath tissues. The black line indicates the veins across the sheath, ligule and blade tissues of maize. (**B**) Cell file numbers and the ratio between the cell length and width were calculated in the leaf blade and sheath tissue between two lateral veins. (**C**) The blade and sheath tissues above or below the ligule of the third leaf from seedlings 10 (stage 1), 11 (stage 2), 12 (stage 3) and 13 (stage 4) days after planting were harvested at the same time. The arrowheads point to the leaf ligule. (**D**) Microscopic view of the blade (B), ligule (L) and sheath tissues (S) from stage 0 to stage 4 plants. The arrowheads point to the leaf ligule.

In the mature maize leaf, the proximal sheath and distal blade tissues are separated by the leaf ligule (Figure 1A,D). Relative to the leaf blade, epidermal cells in the sheath tissue are smaller and contain fewer cell files, as determined by counting the number of cell files between the same veins across the sheath and blade (Figure 1A,B). To investigate developmental and maturation changes in the sheath tissue, we planted maize seeds every day for 13 days and then harvested the leaf blades and sheaths from the third leaf on the same day, when seedlings ranged in age from 9 to 13 days after planting in order to minimize the environmental variation at the time of harvest. Two millimeter-wide sections of sheath and blade tissues just below or above the ligule were harvested from the third leaf at 10 (stage 1, S1), 11 (stage 2, S2), 12 (stage 3, S3), and 13 (stage 4, S4) days after planting. The sheath tissue was poorly defined on the third leaf at 9 days after planting (stage 0), so we could not harvest tissue from this stage (Figure 1C,D). Although the specification of sheath cell fate is complete in the leaf primordium [16], the sheath tissues we harvested from stage 1 to 4 were still undergoing differentiation and maturation (Figure 1C,D). In total, 221.9 million high-quality reads were obtained from the RNA-seq analysis, of which 164.0 million (73.9%) were uniquely mapped to the maize reference genome. We detected 24,704, 25,225, 25,224, and 24,498 expressed genes from stages 1 to 4, respectively, and 23,557 genes were expressed in all four stages during sheath maturation. 

### 2.2. Maturation Gradient in the Leaf Sheath of Maize

**Figure 2:**
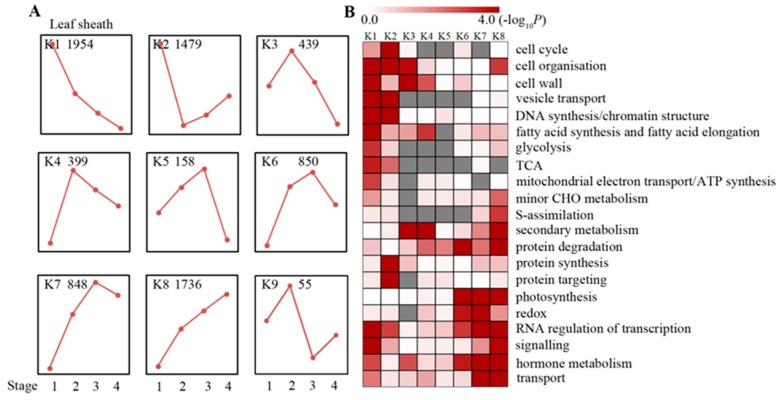
Transcriptome profiling during sheath maturation in maize. (**A**) K-means clustering showing the expression profile of the maize sheath transcriptome. Eight major clusters (K1–K8) were identified along the four developmental stages (stage 1 to 4) in sheath tissues from 7918 differentially expressed genes. (**B**) Functional category enrichment was calculated using the MapMan binning method among the eight major clusters in A. The shade of red represents the significance level of log 10 transformed *p*-values calculated from Fisher’s exact test. Gray blocks indicate functional category enrichment was absent.

We identified 7918 differentially expressed (DE) genes in the leaf sheath at four maturation stages with a false discovery rate (FDR) controlled at 0.001 (Supplemental Table S1), which accounted for approximately 31% of the sheath transcriptome. We then assigned genes to functional categories and grouped the genes by developmental dynamics using the k-means clustering algorithm. We identified nine clusters (K1–K9; Figure 2; Supplemental Figure S1 and Supplemental Table S1) from the leaf sheath and eight main clusters (K1–K8) that accounted for approximately 99% of the DE genes in four stages of development and maturation. As shown in Figure 2A,B, genes encoding proteins involved in cellular organization (e.g., actin and tubulin), vesicular transport, (e.g., syntaxin and snare), and DNA synthesis/chromatin structure were greatly enriched in clusters K1 and K2 and represented genes required for the installation of the basic cellular infrastructure. These genes are expressed at their highest levels in the early stage of sheath maturation (stage 1). Differences also existed between the K1 and K2 clusters. Namely, genes encoding fatty acid synthesis and elongation-related products, such as 3-ketoacyl-CoA synthase 4, 3-ketoacyl-CoA synthase 6, 3-ketoacyl-CoA synthase 9, 3-ketoacyl-CoA synthase 11 and 3-ketoacyl-CoA synthase 20 were differentially expressed [17]. The expression of genes related to respiration (including glycolysis, the tricarboxylic acid cycle and mitochondrial electron transport); signaling genes, especially G-proteins and LRR receptor kinases; and some transcription factors were greatly enriched in cluster K1 and slightly enriched in cluster K2, and genes involved in protein synthesis and targeting were only enriched in K2. The genes in clusters K3 and K4 were expressed at their highest levels in stage 2 and mainly participate in cell wall biosynthesis/modification and secondary metabolism. The genes that were highly expressed in stage 3 were included in clusters K5–K7 and are mainly involved in pathways related to photosynthesis (K6 and K7), hormone metabolism (K6 and K7), and redox and transcription regulation (K6 and K7), indicating that the sheath tissues at stage 3 begin building photosynthetic machinery. The genes in the K8 cluster were enriched in the functional categories of photosynthesis, sulfate assimilation, and secondary metabolism, e.g., lignin biosynthesis to strengthen the sheath, as well as cell organization, especially actin and fibrillin family proteins that are required for plastoglobule development. This highlighted the functions of both photosynthesis and plant strengthening in the mature sheath of maize. Taken together, these data revealed metabolic changes during the maturation of the sheath tissue in the maize leaf.

### 2.3. Cell Wall and Lignin Synthesis during Leaf Sheath Maturation in Maize

**Figure 3:**
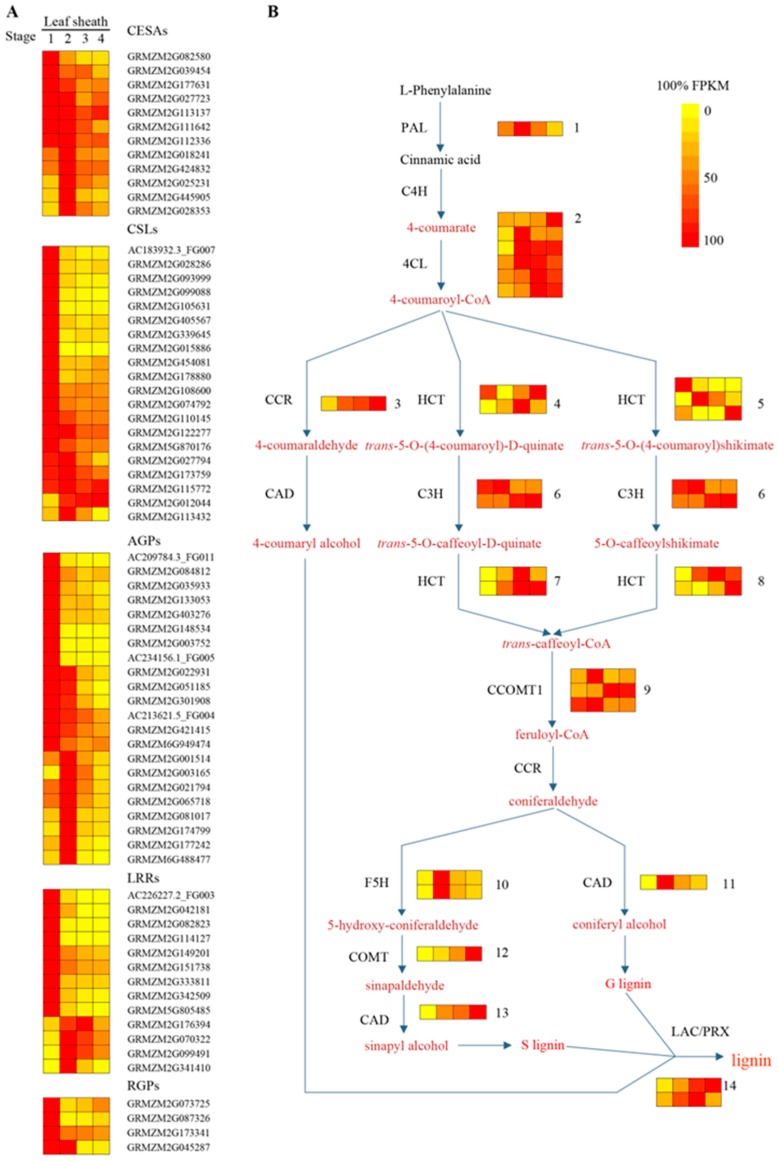
Heatmaps showing the expression profiles of genes related to cell wall (**A**) and lignin biosynthesis (**B**). Relative gene expression was calculated from the maximum fragments per kilobase million (FPKM) values among the maturation zones. The detailed expression patterns and identities of the genes in each of these biosynthetic pathways are shown in Supplementary Table S2.

The cell wall plays an important role in shaping and strengthening cells. We noticed a dramatic change in the expression of cell wall-related genes during sheath maturation (Figure 3A). As shown in Figure 3A, cellulose synthases (*CESA*s)—the key genes of cellulose biosynthesis in primary and secondary cell walls—exhibited the highest expression levels in stages 1 or 2. Most of them exhibited dramatically decreased expression in stages 3 and 4, during late sheath maturation. Consistent with the expression patterns of the *CESA*s, most genes encoding CSL (cellulose synthase-like) proteins and cell wall proteins—for example, arabinogalactan proteins (AGP), leucine-rich repeat family proteins (LRR), and reversibly glycosylated polypeptide (RGP)—also showed decreased expression patterns in the late stages (S3 and S4) of sheath maturation. This indicated that the cell wall might be built in stages 1 and 2.

As a main component of the secondary cell wall, lignin plays an important role in providing strength and rigidity to support the cells and the plant body. As shown in Figure 3B (Supplemental Table S2), key enzymes related to lignin biosynthesis [18,19]—e.g., 4-coumarate-CoA ligase (4CL), cinnamoyl CoA reductase (CCR), *O*-methyltransferase (COMT), and cinnamyl alcohol dehydrogenase (CAD)—exhibited increased expression during the maturation of the sheath, which indicated the accumulation of lignin with the maturation of the leaf sheath tissues.

### 2.4. Changes in Transcription Factors during Sheath Maturation in Maize

**Figure 4:**
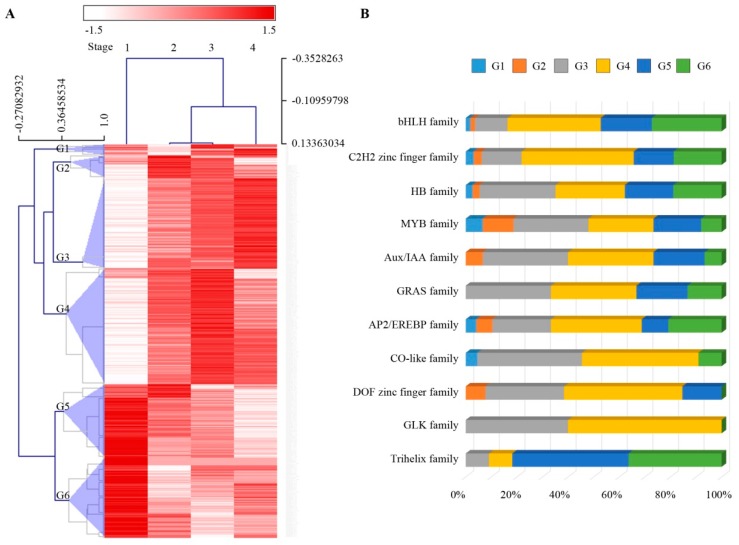
Dynamics of accumulated transcription factor profiles. (**A**) Hierarchical clustering (HCL) of the transcription factors related to sheath maturation in maize, six lineages (G1 to G6) were identified. The bar represents the expression of transcription factors normalized by row across four stages during sheath maturation in the HCL program. (**B**) Distribution of transcription factor families in groups G1 to G6 in the leaf sheath.

As key factors that regulate gene expression, transcription factors (TFs) play important roles in plant growth, development and the response to various environmental stress. We detected 456 differentially expressed TFs (*q* < 0.001) during sheath maturation and grouped them into six groups (G1 to G6, Figure 4A and Supplemental Table S3) using a hierarchical clustering (HCL) program. As shown in Figure 4A, groups G3 to G6 included most of the TFs (91%). The TFs in group G3 exhibited their highest expression levels in stage 4 during sheath maturation, and those in G4 were expressed at their peak level in stage 3. The goladen2-like (GLK) family of TFs, including *GLK1*, which regulates chloroplast development in maize [20], were exclusively enriched in groups G3 and G4 (Figure 4B). The DNA binding with one finger (DOF) family of TFs, such as *CDF3*, likely regulate photoperiod gene expression [21]. CO-like family members, such as *COL3* [22,23], regulate gene expression in photomorphogenesis and during lateral root development and function as a day length-sensitive regulator of shoot branching. The MYB family of TFs, such as *MYB4* [24,25], which respond to UV-B and *LHY* (involved in circadian rhythm [26]), were all mainly enriched in groups G3 and G4. As such, they indicated an increase in photosynthesis in stage 3 and 4 sheath tissues. Groups G5 and G6 included TFs that were highly expressed in stage 1, the earliest maturation stage that we harvested. The trihelix family members were highly enriched in both groups G5 and G6, and the basic helix-loop-helix (bHLH) family (e.g., *MUTE*) controlled meristemoid differentiation during the early stage of stomatal development [27]. The changes in the expression of TFs suggested the transcriptional regulation of gene expression during sheath maturation.

### 2.5. Identification of Genes Expressed at High Levels in the Leaf Sheath

To determine whether some genes were specifically expressed in the leaf sheath, we compared the sheath transcriptome with that of the blade at each stage during sheath maturation. In total, 167 genes were expressed at higher levels and 362 genes were expressed at lower levels in the sheath, than that in the blade, across the four maturation stages, with the FDR controlled at 0.001 (Supplemental Table S4).

**Figure 5:**
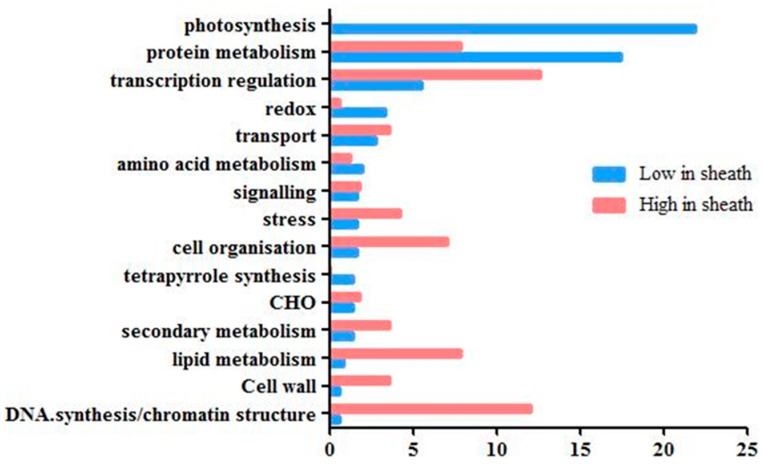
Functional category distribution of genes expressed at high or low levels in the sheath tissue.

As shown in Figure 5, of the 362 genes that were expressed at lower levels in the sheath than in the blade, 22% of them participated in the photosynthetic pathway. In contrast, of the 167 genes that were expressed at higher levels in the sheath than in the blade, none participated in photosynthesis. In addition to the photosynthetic pathway, genes involved in tetrapyrrole synthesis and redox scavenging were also specifically expressed at high levels in the leaf blade, consistent with the function of the green leaf blade to harvest light for photosynthesis, and redox reactive species generated by the light need to be controlled by enzymes such as ascorbate peroxidase. Interestingly, for genes in the functional categories of DNA synthesis/chromatin structure, cell wall development, lipid metabolism and cell organization, the enriched genes were expressed at much higher levels in the leaf sheath than in the blade. This may reflect a prolonged phase of cell growth and division in sheath tissues relative to the blade. As almost no sheath-specific genes were reported in a previous study on maize, we focused on identifying genes that were uniquely expressed at high levels in the leaf sheath.

As shown in Table 1, we identified 15 genes that were expressed at very low levels or not significant levels in the leaf blade, while their expression was high in the leaf sheath across the four developmental stages sampled. Among them were two genes (*GRMZM2G026556* and *GRMZM2G022606*) that are homologous to *BLADE ON PETIOLE2* (*BOP2*) in *Arabidopsis* and rice. BOP2 acts in cells adjacent to lateral organ boundaries to repress genes that confer meristem cell fate and induce genes that promote lateral organ fate and polarity in *Arabidopsis*, and plays a primary role in rice to regulate the proximal-distal axis [28,29]. *DON-glucosyltransferase 1* (*DOGT1*, *GRMZM2G403740*), encodes an enzyme that presumably regulates BR activity in *Arabidopsis* [30,31], and a homolog of *SMALLER WITH VARIABLE BRANCHES* (*SVB*, *GRMZM2G131409*), a protein with a conserved domain of unknown function (DUF538) that influences trichome development in *Arabidopsis* [32] were also expressed at higher levels in sheath relative to blade. In addition, *glycerol-3-phosphate acyltransferase* (*GPAT2*, *GRMZM2G033767*) and GDSL-like Lipase/Acylhydrolase superfamily protein (*GDSL-like Lipase*, *GRMZM5G862317*), which likely participates in lipid metabolism [33,34]; *carboxyesterase* (*CXE18*, *GRMZM2G104141*), which may play a role in cell elongation [35] and senescence-associated gene (*SAG12*, *GRMZM2G061879*), which controls nitrogen allocation during senescence in *Arabidopsis* [36], were all highly expressed in the sheath tissue. This indicated the possible role of these genes in sheath development and maturation.

Transcription factors may also play important roles in distinguishing sheath tissues from blade tissues. The B3 domain-containing transcription factor *NGA1*, which negatively regulates cell proliferation in *Brassica rapa* [37,38], is expressed at a high level in the sheath (*GRMZM2G082227*) tissue only, which may suggest its role in prohibiting the development of sheath tissue into blade tissue. The maize *NTT* (*NO TRANSMITTING TRACT*) homologs *GRMZM2G071101* and *GRMZM2G445684*, two members of the C2H2-type zinc finger family that determine the distal cell fate in the root and influence transmitting tract development and pollen tube growth in *Arabidopsis* [39,40,41], as well as the homeobox family member *GRMZM2G034113*, the homolog of which (the *AtHB7* gene) is involved in ABA signaling during water stress, were all highly expressed in the sheath tissue and exhibited quite low expression in the blade [42,43]. This suggested that these TFs may play a role in the development or tissue-specific function of the leaf sheath. To confirm the reliability of the RNA-seq data, the expression levels of these four transcription factors at the four blades and sheath maturation points discussed above were also quantified by qRT-PCR (Supplemental Figure S2, Supplemental Table S5). The consistency between the results of the two methods verified the reliability of the enriched expression of these TFs in the leaf sheath.

## 3. Discussion

Cereal crops, such as maize, are a primary calorie source for human and animal diets, and the most important organs for the conversion of photosynthetic energy into carbon are the leaves. Therefore, understanding leaf development in maize is important for agriculture to further increase maize yields. Several studies have uncovered photosynthetic development in the maize leaf blade [6,7]. However, very few studies have focused on leaf sheath tissues. Consequently, very few sheath-specific genes in maize have been identified and our understanding of sheath development is also limited. 

Using a comprehensive transcriptome dataset, we captured the dynamic transcriptome changes that occurred during leaf sheath tissue maturation. In the earliest stage of maturation (stage 1), basic cellular structures were constructed, as the genes associated with cell organization, vesicle transport and DNA synthesis/chromatin structure were highly expressed. In stage 2, as the sheath tissue continued to expand, there was an increase in the expression of the genes required for cell wall biosynthesis, modification, and secondary metabolism. By stage 3, the sheath cells were well primed for photosynthesis, as they expressed genes required for photosynthesis and redox reactions at high levels. In addition to genes required for photosynthesis, by stage 4, there was an increased expression of genes participating in secondary metabolism and lignin biosynthesis, which suggests an increase in the strength of the sheath tissues. Therefore, these four stages of leaf sheath differentiation define distinct steps in the sheath maturation process. 

We inferred from the RNA-seq data that different tissues in the maize leaf are highly specialized in their biological functions. By comparing the transcriptome of the leaf blade and sheath and identifying the genes expressed at high or very low levels in the sheath tissue across four stages of maturation, we found that increased expression of photosynthesis-related genes in the leaf blade corresponded with its exclusive function in photosynthesis. We also found increased expression of genes that participate in DNA synthesis/chromatin structure, cell wall maintenance, lipid metabolism and cell organization in the sheath tissue, which are involved in building and strengthening the tissue. Interestingly, several genes were highly expressed, specifically in the sheath tissue (e.g., the *BOP2* homologs *GRMZM2G026556* and *GRMZM2G022606*). The *BOP* gene is required for the patterning of the leaf petiole and blade in *Arabidopsis* [28], and a recent study demonstrated that it activates proximal sheath differentiation and suppresses distal blade differentiation in rice [29], indicating its important role in sheath development. More research needs to be performed to test the functions of *DOGT1* (*GRMZM2G403740*) and *SVB* (*GRMZM2G131409*) in maize, even though their *Arabidopsis* homologs affect hormone activity and influence plant development [30,31,32].

In our survey, we found that TFs may play important roles in both sheath development and functional specialization. During the early stage of sheath maturation, trihelix family and bHLH family members may participate in cell development, and in the late stage of maturation. When the sheath starts to carry out photosynthesis, the members of the GLK, DOF and CO-like families may play roles in light signaling and chloroplast development. Interestingly, TFs from the B3 domain (*GRMZM2G082227*), C2H2-type zinc finger (*GRMZM2G071101* and *GRMZM2G445684*) and homeobox (*GRMZM2G034113*) families were expressed at a higher level in the sheath tissue than in the blade, which may suggest a role for these transcription factors in sheath development and functional specialization. Taken together, our results provide detailed insight into dynamic changes during maize leaf sheath maturation and serve as a valuable resource for maize functional genomics study.

## 4. Materials and Methods

### 4.1. Plant Material and Sampling

Maize inbred B73 were planted in the growth chamber every day for 13 days with a light intensity of 500 µmol/m^2^/s, 12:12 L/D, 31 °C L/22 °C D, and 50% relative humidity. The first planting was 13 days, and the blade and sheath of the third leaves were harvested at 2 mm sections above and below the leaf ligule from the seedlings on the 10th (stage 1), 11th (stage 2), 12th (stage 3) and 13th (stage 4) days after planting. Tissue from 10 leaves was harvested as a pool and frozen in liquid nitrogen for RNA extraction and library construction, and 3 biological replicates were harvested at each stage.

### 4.2. SEM Observation

Scanning electron microscopy was used to observe the cell structure in the maize leaf blade and sheath tissues. Stage 4 seedlings, including the leaf blade, ligule and sheath were fixed in 4% glutaraldehyde in 25 mM sodium phosphate buffer (pH 6.8) at 4  °C for at least 24  h. Then, the samples were rinsed by 25 mM sodium phosphate (pH 6.8) and dehydrated in a graded ethanol series at 4 °C. Thereafter, the samples were immersed in tertiary butyl alcohol and stored in a refrigerator at 4 °C. After sublimation of the frozen tertiary butyl alcohol in a vacuum, the samples were mounted on SEM stubs with double-sided tape, sputter-coated with gold, and examined under a scanning electron microscope (JSM-6610LV, JEOL, Tokyo, Japan).

### 4.3. RNA-Seq Library Construction and Sequencing

Total RNA was extracted from each sample using the TRIZOL reagent (Invitrogen, Carlsbad, CA, USA). The concentration of the RNA was determined using a DeNovix Spectrophotometer (DeNovix, DS-11, Wilmington, DE, USA), and the RNA quality was determined by 1% agarose gel electrophoresis. The RNA-Seq library construction method of Wang et al. was used [44]. Briefly, the total RNA was purified using the TURBO DNA-free Kit (Ambion, Austin, TX, USA) to completely remove genomic DNA contamination, after that, the poly(A) RNA was isolated from the purified total RNA using poly(T) oligonucleotide-attached magnetic beads (Invitrogen, Carlsbad, CA, USA). Following purification, the mRNA was fragmented into small pieces using divalent cations under elevated temperatures, and the cleaved RNA fragments were reverse-transcribed into first-strand cDNA using reverse transcriptase and random primers. Second-strand cDNA synthesis was performed using DNA polymerase I and RNase H, and the cDNA fragments were processed for end repair, a single adenine base was added, and sequences were ligated to the adapters. These products were then purified and enriched by PCR to create the final cDNA libraries and sequenced on the Illumina Hi-Seq 2500 for single-ended sequencing. Sequencing data have been deposited in the NCBI Sequence Read Archive under the accession number SRP133466.

### 4.4. Mapping of Reads

The FASTX-toolkit (http://hannonlab.cshl.edu/fastx_toolkit/) was used to remove the adapters of the raw reads with the fastx_clipper setting a parameter of “-a ADAPTER”. The sequence quality was examined by FastQC (http://www.bioinformatics.babraham.ac.uk/projects/fastqc/) setting “-t 6 –noextract”, and low quality reads were filtered by the FASTX-toolkit setting parameters as “q20p80” (i.e., for each read kept, 80% of bases must have sequence quality greater than 20, which indicates 1% sequencing error rate). The clean reads were mapped to the Maize B73 genome (version 3) obtained from Phytozome using Tophat v2.0.10 (http://tophat.cbcb.umd.edu/) setting -read-mismatches 2 --num-threads 6’. Count data were generated by Cuffdiff embed in Cufflinks pipeline v2.1.1 setting “--num-threads 6 -frag-bias-correct” [45]. The gene expression level was normalized as FPKM, and genes with FPKM > 1 were considered to be expressed. 

### 4.5. Defining Differentially Expressed Genes and Cluster Analysis

Differentially expressed (DE) genes were identified by DESeq v1.34.1 [46] in Bioconductor (http://www.bioconductor.org/), based on a comparison across all the sheath samples (pairwise comparisons with stage 1 as control) or between the blade and sheath in each stage with a false discovery rate (FDR) controlled by the Benjamini and Hochberg method set at 0.001. The MapMan program (http://mapman.gabipd.org) was used to assign genes into functional categories. Clustering analysis of KMC (k-means clustering)—based on the Kendall’s Tau distance metric with maximum iterations set as 50—and HCL support trees, based on Kendall’s Tau distance metric with average linkage method, was performed through the MEV software (http://www.tm4.org/). Functional enrichment analysis was performed using Fisher’s exact test, according to the method by Li et al. [6]. The ID of the DE genes were used to generate a text file. Then, the R software was used to complete the enrichment analysis.

### 4.6. Quantitative RT-PCR Analysis

cDNA was prepared using the EasyScript One-Step gDNA Removal and cDNA Synthesis SuperMix (TRAN, Beijing, China) and qRT–PCR analyses were conducted using TransStart Tip Green qPCR SuperMix (TRAN, Beijing, China) on a Step One System (Applied Biosystems, Branchburg, NJ, USA). The quantification method (2^−∆∆Ct^) was used and the variation in expression was estimated using three biological replicates [47]. The maize *Ubi2* (UniProtKB/TrEMBL, Q42415, https://www.uniprot.org/statistics/TrEMBL) gene was used as an internal control to normalize the data. The PCR conditions consisted of an initial denaturation step at 94 °C for 30 s, followed by 40 cycles at 94 °C for 5 s and 60 °C for 30 s.

## Figures and Tables

**Table 1:** Genes expressed at high levels in the sheath tissue.

			Blade				Sheath			
ID	Short Description	Symbol	Stage				Stage			
			1	2	3	4	1	2	3	4
GRMZM2G026556	BTB/POZ domain-containing protein	BOP2	0.3	0.28	1.05	0.73	9.91	18.47	31.56	38.95
GRMZM2G022606	BTB/POZ domain-containing protein	BOP2	0.4	0.3	0.55	0.3	9.09	10.58	14.64	20.82
GRMZM2G403740	don-glucosyltransferase 1	DOGT1	0.05	0.06	0.55	0.11	0.37	2.12	3.89	13.76
GRMZM2G131409	Encodes Smaller with Variable Branches	SVB	0.58	0.52	0.33	0	0.49	15.39	16.42	8.07
GRMZM2G033767	glycerol-3-phosphate acyltransferase 2	GPAT2	0	0.55	1.25	0.6	0.83	63.1	105.91	33.77
GRMZM5G862317	GDSL-like Lipase/Acylhydrolase superfamily protein	GDSL-like Lipase	0.51	2.79	3.18	3.56	57.19	42.25	50.57	39.53
GRMZM2G104141	carboxyesterase 18	CXE18	0	0.72	2.11	0.87	0	21.06	73.57	39.17
GRMZM2G061879	senescence-associated gene 12	SAG12	1.41	6.92	7.68	1.38	1.58	51.99	136.22	196.46
GRMZM2G082227	AP2/B3-like transcriptional factor family protein NGATHA1	NGA1	1.51	2.08	2.48	2.4	7.13	6.81	14.38	16.34
GRMZM2G445684	C2H2-type zinc finger family protein No Transmitting Tract	NTT	0.22	0.17	0.13	0.16	17.68	15.79	39.79	42.81
GRMZM2G071101	C2H2-type zinc finger family protein No Transmitting Tract	NTT	0.31	1.77	0.75	0.34	10.08	19.09	45.68	42.21
GRMZM2G034113	homeobox 7	HB-7	1.67	0.91	1.97	6.79	14.24	17.7	43.08	43.7
GRMZM5G842695	MATE efflux family protein	-	0.11	0.6	0.38	0.12	0.12	3.07	5.09	6.33
GRMZM2G343291	Protein of unknown function	-	4.47	13.77	4.61	0.57	2.06	69.06	117.95	94.52
GRMZM2G136571	alpha/beta-Hydrolases superfamily protein	-	0.83	0.77	1.53	0.55	0.63	7.03	34.12	25.2
GRMZM2G412436	Protein of unknown function	-	0.98	2.39	1.31	0.17	2.25	18.56	15.16	4.33

Note: The expression of genes was represented by the FPKM value in the table.

## Supplementary Materials

The following are available online at https://www.mdpi.com/1422-0067/20/10/2472/s1. Figure S1. The original graphs showing the K-mean clusters of the differentially expressed genes (DEGs); Figure S2. qRT-PCR and RNA-seq comparison; Table S1. K-mean clusters of DEGs during sheath maturation; Table S2. Expression of genes involved in cell wall and lignin synthesis; Table S3. Hierarchical clustering (HCL) of transcription factors related to sheath maturation in maize; Table S4. Genes expressed at higher or lower levels in the sheath tissue compared with their expression in the blade tissue in maize across four stages of maturation (FDR < 0.001); Table S5. Primers used for qRT-PCR.
