# Supplementary figures and images for: Transcriptomic Analysis of Leaf Sheath Maturation in Maize
Source: Int J Mol Sci. 2019 May 19;20(10):2472. doi: 10.3390/ijms20102472 (PMC6566692; doi:10.3390/ijms20102472)

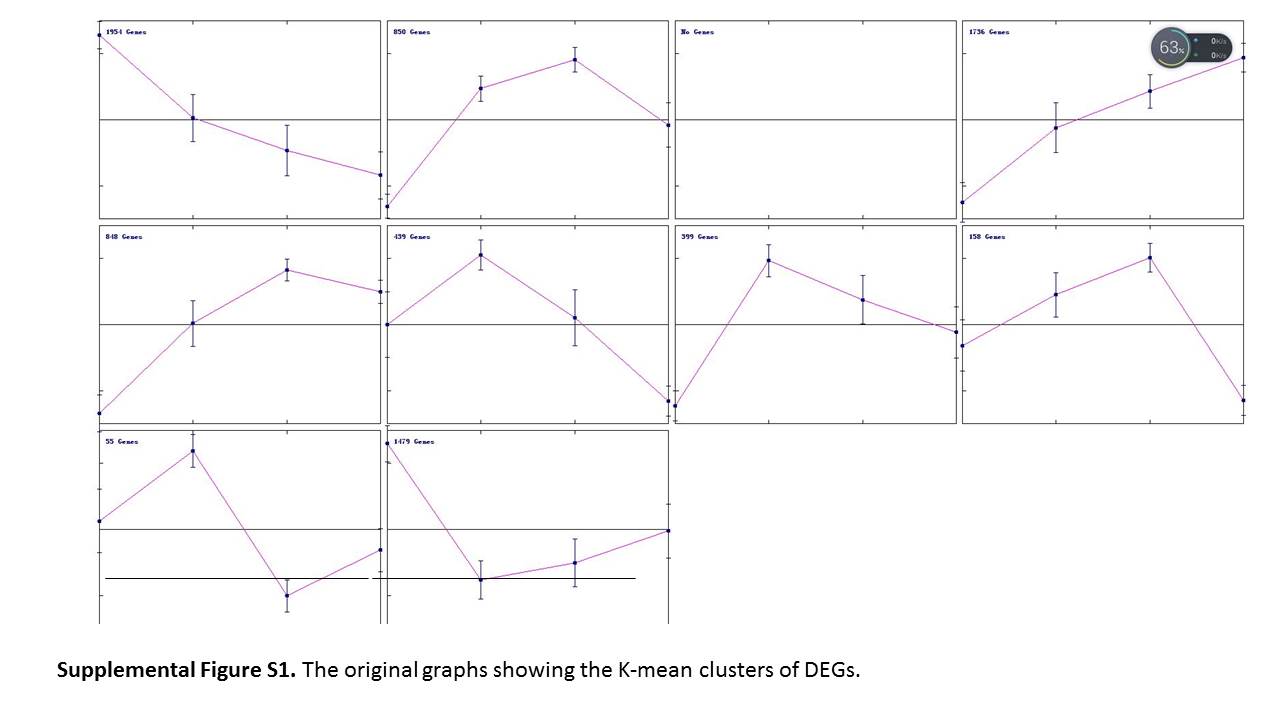


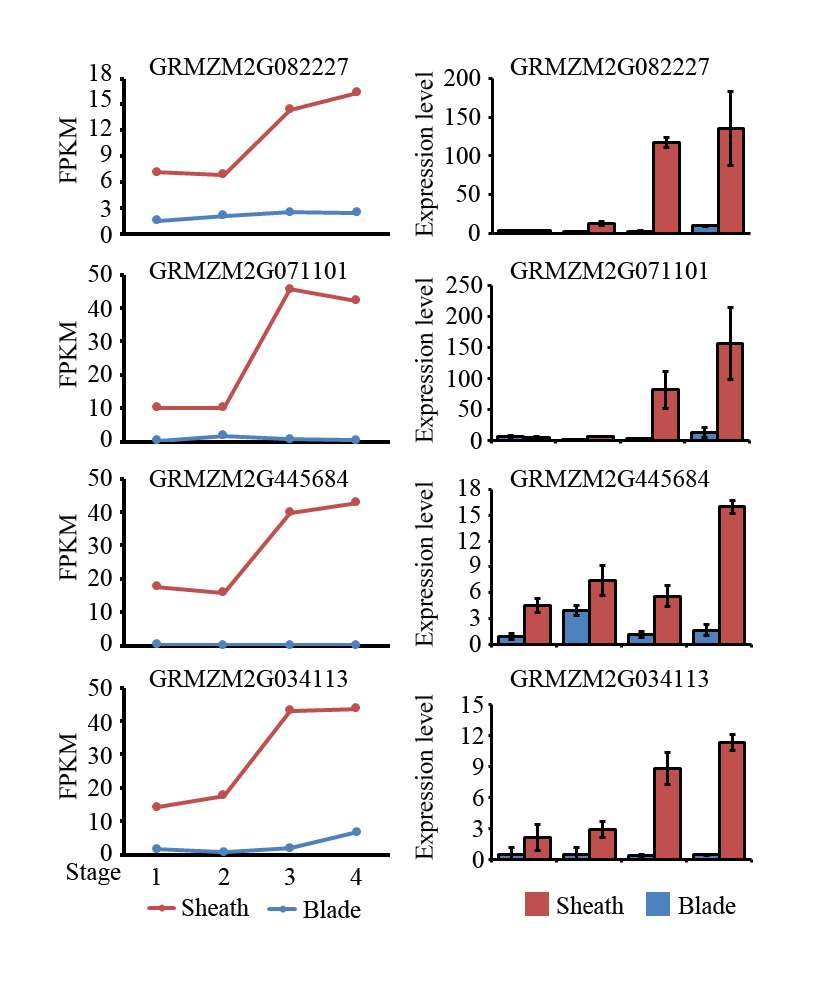


**Figure S2**. qRT-PCR and RNA-seq comparison.

Supplement: Supplementary file 1 [file ijms-20-02472-s001.zip › Supplementary data/ijms-487857 supplementary materials.docx]
